# The screening for anticoagulant rodenticide gene *VKORC1* polymorphism in the rat *Rattus norvegicus, Rattus tanezumi* and *Rattus losea* in Hong Kong

**DOI:** 10.1038/s41598-022-16550-3

**Published:** 2022-07-22

**Authors:** Elaine Y. Y. Huang, Sean T. S. Law, Wenyan Nong, Ho Yin Yip, Theethawat Uea-Anuwong, Ioannis Magouras, Jerome H. L. Hui

**Affiliations:** 1 School of Life Sciences, Simon F.S. Li Marine Science Laboratory, State Key Laboratory of Agrobiotechnology, The Chinese University of Hong Kong, Hong Kong, China; 2 Department of Infectious Diseases and Public Health, City University of Hong Kong, Hong Kong, China

**Keywords:** Molecular biology, Zoology

## Abstract

Anticoagulants are a major component of rodenticides used worldwide, which function by effectively blocking the vitamin K cycle in rodents. The rat Vitamin K epoxide Reductase Complex (VKORC) subunit 1 is the enzyme responsible for recycling vitamin K, and five substitution mutations (*Tyr139Cys, Tyr139Ser, Tyr139Phe and Leu128Gln* and *Leu120Gln*) located in the *VKORC1* could result in resistance to anticoagulant rodenticides. This study carried out a *VKORC1-*based survey to estimate the anticoagulant rodenticide resistance in three *Rattus* species (*R. losea*, *R. norvegicus*, and *R. tanezumi*) collected in Hong Kong. A total of 202 rats captured in Hong Kong between 2017 and 2021 were analysed. Sequencing of molecular marker cytochrome c oxidase subunit 1 (COX1) was carried out to assist the species identification, and the identities of 52 lesser ricefield rats (*R. losea*), 81 common rats (*R. norvegicus*) and 69 house rats (*R. tanezumi*) were confirmed. Three *VKORC1* exons were amplified from individuals by PCR followed by Sanger sequencing. A total of 47 *R. tanezumi* (68.1%) contained *Tyr139Cys* mutation in *VKORC1* gene, and half of them were homozygous. None of the collected *R. losea* and *R. norvegicus* were detected with the five known substitutions leading to anticoagulant rodenticides resistance, and previously undescribed missense mutations were revealed in each species. Whole genome sequencing was further carried out on some individuals, and single nucleotide polymorphisms (SNPs) were also identified in the introns. This is the first study investigating the situation of anticoagulant rodenticide resistance in the rats collected in Hong Kong. Given that the efficacy of rodenticides is crucial for effective rodent management, regular genetic testing as well as population genomic analyses will be required to both monitor the situation and understand the adaption of different rat haplotypes for integrated pest management. Susceptibility tests for individual rodenticides should also be conducted regularly to assess their effectiveness on local species.

## Introduction

Rodents have been generally regarded as pests as they cause economic losses and transmit rodent-borne diseases^1,2^. In Hong Kong, eight species of rats and mice had been previously identified including *Bandicota indica*, *Mus caroli*, *M. musculus*, *Niviventer fulvescens*, *Rattus norvegicus*, *R. rattus*, *R. tanezumi*, and *R. sikkimensis*^3^. In recent, the first ever reported transmission of rat hepatitis E virus species C genotype 1 to human had also been identified in Hong Kong^4^. This emphasizes the importance of maintaining efficient rodent control in order to safeguard public health.

Anticoagulant pesticides are commonly used in agricultural and urban rodent controls since few decades ago^5,6^. The anticoagulant rodenticides including warfarin and coumarin derivatives function effectively via binding with the vitamin K epoxide reductase of the rodents^7–9^. Resistance to several anticoagulant rodenticides including has been reported worldwide since 1960s^10–13^, and the vitamin recycling gene *Vitamin K epoxide reductase complex subunit 1* (*VKORC1*) is now known to associate with the anticoagulant rodenticides-resistance^14–16^.

Studying mutations of the exonic nucleotide composition or single nucleotide polymorphisms (SNPs) of *VKORC1* gene provides crucial information on resistance to As rodenticides and efficacy of pest control^17–19^. For instance, ~ 70% of sampled common or Norwegian rats (*R. norvegicus)* in the United Kingdom carried one of the five known missense mutations (*Tyr139Cys, Tyr139Ser, Tyr139Phe and Leu128Gln* and *Leu120Gln)*^20^, while these mutations could confer certain level of resistance to both first and second generation of anticoagulant rodenticides^16,17,19^. In a recent *VKORC1-*based SNP survey in mice and rats in the United States, it has also been suggested that resistances detected in the 1980s were likely due to mutations of *Leu128Ser* and *Tyr139Cys* in house mice (*M. musculus domesticus)*, *Arg35Pro* in common or Norwegian rats (*R. norvegicus*), and *Tyr25Phe* in roof rats (*R. rattus*)^21^. Nevertheless, limited information was obtained from Asia, including Hong Kong. We therefore collected rodents from Hong Kong and carried out a *VKORC1-*based survey to estimate the anticoagulant rodenticide resistance situation that could compromise pest management.

## Materials and methods

### Sampling and DNA extraction

A total of 202 tail samples from dead rodents were provided to The Chinese University of Hong Kong by the Food and Environmental Hygiene Department, The Government of the Hong Kong Special Administrative Region and the City University of Hong Kong. The rodents were captured using traps from different locations in Hong Kong between 2017 and 2021. Tail samples were stored at − 20 °C before further experimental procedures. Genomic DNA extraction was carried out using QIAamp DNA mini kit (QIAgen, Germany) following the manufacturer’s instructions. In brief, 0.02 g of tail tissue were homogenized and incubated with proteinase K at 55 °C for 2 h. The quantity and quality of DNA were determined by Nanodrop (Ratio of 260/280 ~ 1.8 and 260/230 ~ 1.8–2.0) and gel electrophoresis under Gel Doc™ EZ imager (Bio-Rad), respectively.

### Species identification

Molecular identification was carried out via the polymerase chain reaction (PCR) of mitochondrial DNA cytochrome c oxidase subunit 1 (*COX1*) gene using a model of T100™ thermocycler (Bio-Rad). *COX1* gene was amplified using rodent specific primer BatL5310 (5′‐CCT ACT CRG CCA TTT TAC CTA TG‐3′) and R6036R (5′-ACT TCT GGG TGT CCA AAG AAT CA‐3′)^22^ with following parameters: 3 min of denaturation at 95 °C,39 cycles of 30 s at 95 °C, 30 s at 57 °C, and 40 s at 72 °C; and 5 min of final extension at 72 °C. Each reaction consisted of DNA sample (~ 10–20 ng), 1 × PCR buffer, 0.8 mM of dNTPs, 1.5 mM of MgCl_2_, 0.4 μM of each forward and reverse primers, 11.2 μL of dd H_2_O and 1 unit of Taq DNA polymerase. The amplified products (762 bp) were confirmed on 1% agarose gel stained as well as Sanger sequencing (BGI Genomics Company Hong Kong). Obtained sequences were edited with software SnapGene Viewer, and aligned using MEGA X for phylogenetic analysis (Neighbour-joining method and 1000 bootstrap replications).

### VKORC1 sequence analysis

All three exons of *VKORC1* gene were amplified following a previous study using specific primers: (Exon1 forward: 5′-GTG GCG GGT TCT TCC CTC-3′; Exon 1 reverse: 5′-GAC TCC AAA ATC ATC TGG CAA CC-3′), (Exon 2 forward: 5′-AAG AGT AGG GGAC AAG GTG GC-3′; Exon 2 reverse: 5′-GGG TCA CCA AGA CAT GAG GTG-3′) and (Exon 3 forward: 5′-TTT CAC CAG AAG CAC CTG CTG CC-3′; Exon 3 reverse: 5′-ACA CTT GGG CAA GGC TCA TGT G-3′)^13^. The amplified products were confirmed on 2% agarose gel stained as well as Sanger sequencing (BGI Genomics Company Hong Kong). SNP of each exon sequence was compared to the available sequence from NCBI database (*VKORC1* GenBank accession no. AY423047) with MEGA X software. BlastX searches with adjusted sequences were also carried out to locate any missense mutation. Homozygous and heterozygous genotypes of five published missense mutations on exon 3 were further confirmed on each chromatogram using SnapGene Viewer.

### Genome sequencing of selected individuals

DNA of *R. norvegicus* and *R. tanezumi* from 4 localities including Yuen Long (YL_2, YL_3), Wan Chai (Wch_1, Wch_2), Kwun Tong (KTo_4, KTo_5) and Islands (Is_1, Is_6) were proceeded with low-coverage whole genome sequencing (Table 1). Raw sequenced reads were mapped to the *R. norvegicus* reference genome (GenBank assembly accession: GCF_000001895.5) and SNPs were called with Genome Analysis Toolkit (GATK)^23^. The SNP dataset was annotated with the gene models of the reference assembly using SnpEff^24^. The NGS data have been uploaded to NCBI under the BioProject accession number PRJNA723168.

**Table 1:** Whole genome sequencing data information.

Localities	Samples	No. of reads	No. of bases	Coverage
Islands	Is_1	85,375,522	12,795,390,421	4.46
	Is_5	82,871,652	12,384,207,018	4.31
Kwun Tong	KTo_4	86,039,080	12,884,951,094	4.49
	KTo_5	84,913,562	12,688,470,884	4.42
Wan Chai	Wch_1	106,401,502	15,932,650,971	5.55
	Wch_2	85,521,404	12,800,270,918	4.46
Yuen Long	YL_2	96,202,812	14,415,870,223	5.02
	YL_3	81,822,360	12,233,096,364	4.26

## Results

### *VKORC1* exon 3 of *Rattus losea*, *R. norvegicus*, and *R. tanezumi* in Hong Kong

**Figure 1:**
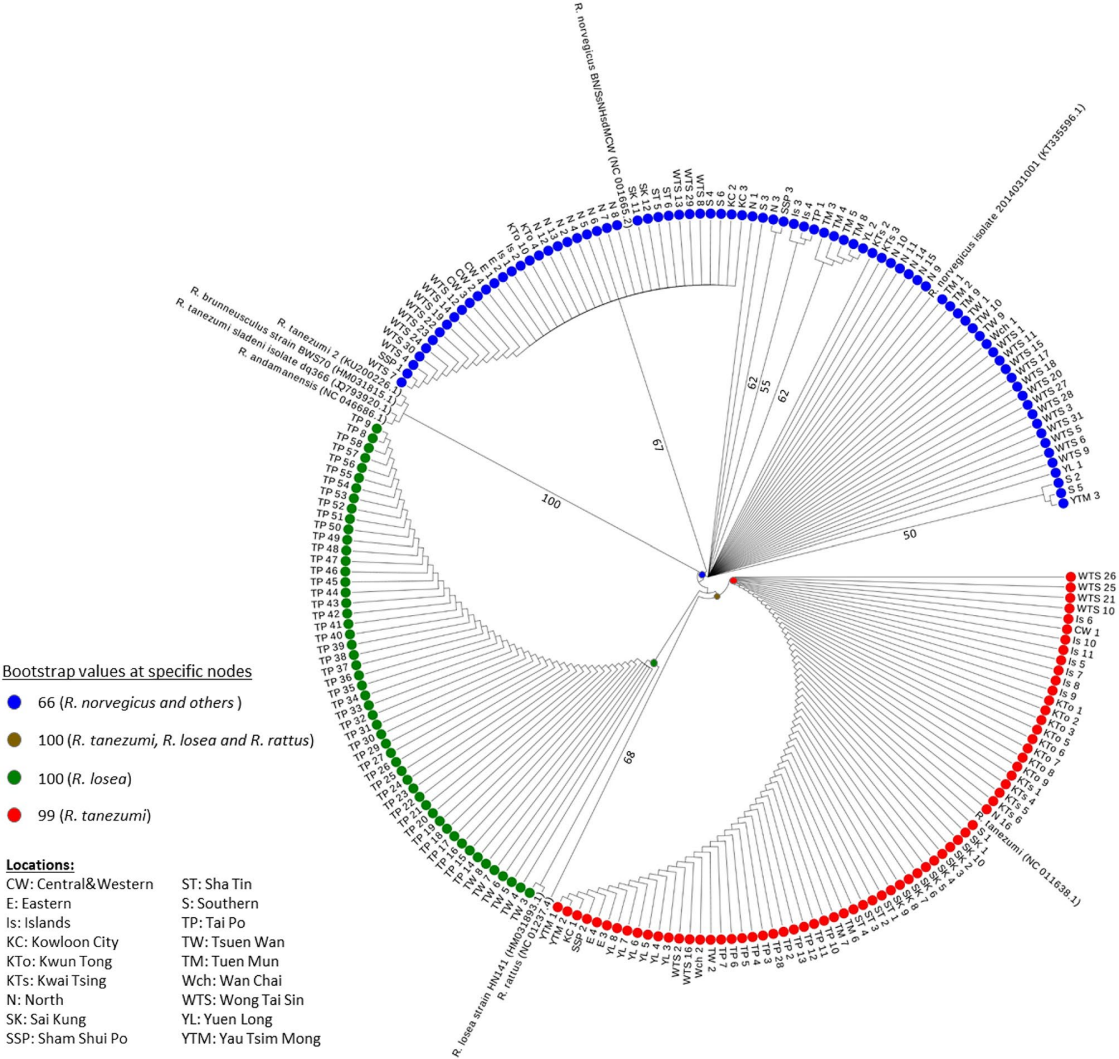
Neighbour-joining tree of rodent’s cytochrome c oxidase subunit 1 (*COX1*) sequences (536 nt). Percentage bootstrap values of nodes that separate the major clades of rodents are indicated in coloured points, while the remaining percentage bootstrap values that are larger than 50 are shown in numbers. Coloured circles at tips represent the rodent species investigated in this study, where blue, red and green correspond to *R. norvegicus*, *R. tanezumi* and *R. losea*, respectively.

In the 202 collected rats, 52, 81, and 69 of them were, *R. losea*, *R. norvegicus*, and *Rattus tanezumi*, respectively (Fig. 1). Greater genetic diversity was also observed in the *COX1* of *R. norvegicus* than the two other captured species.

Among the five previous reported mutations in *VKORC1* exon 3 reported elsewhere in the world (*Tyr139Cys, Tyr139Ser, Tyr139Phe and Leu128Gln* and *Leu120Gln*), only *Tyr139Cys* mutation was found in the *R. tanezumi* samples but not in the other collected species.

**Figure 2:**
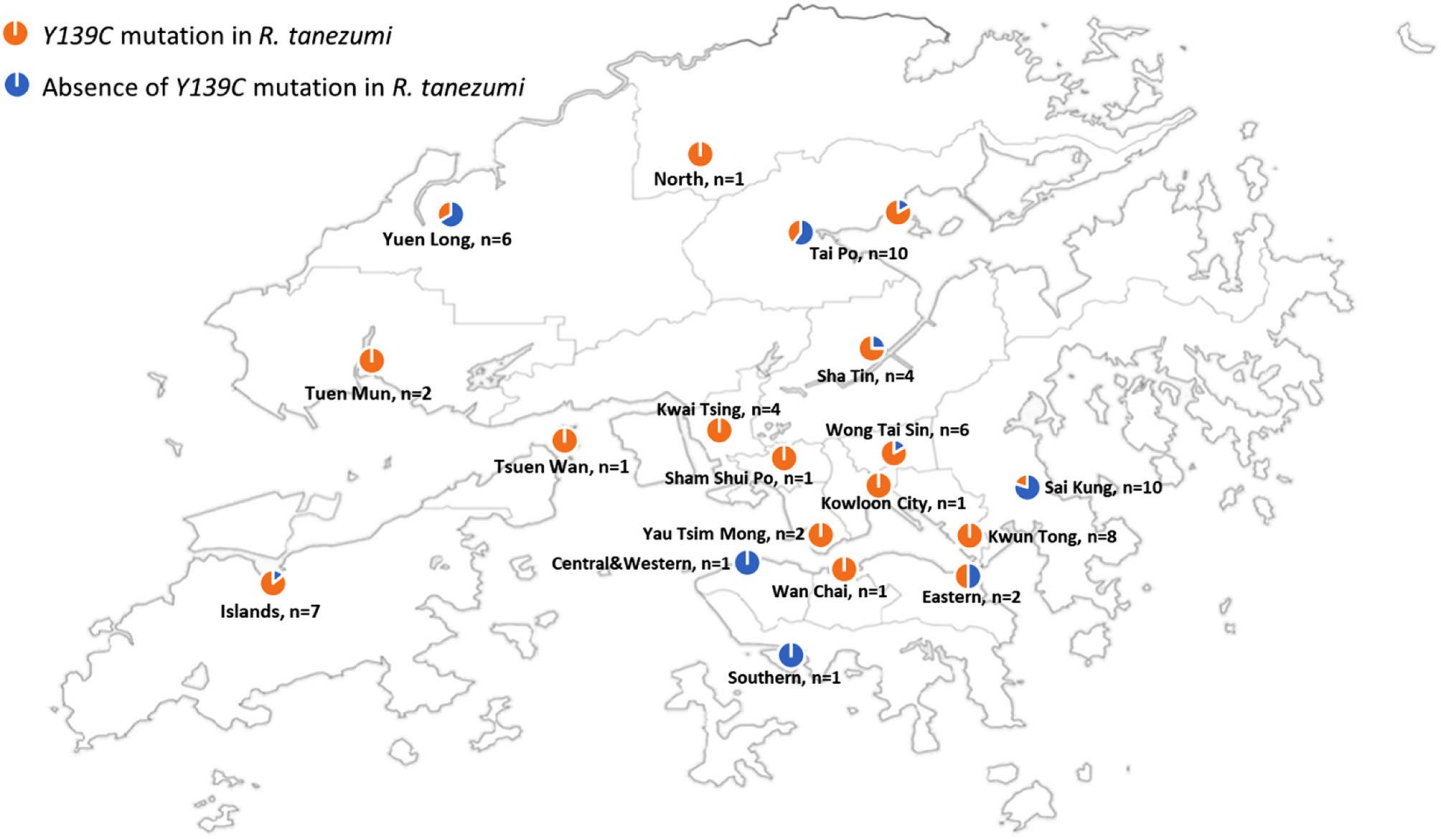
Geographic distribution of *Y139C* mutation found in *R. tanezumi.*

In the 69 collected *R. tanezumi*, 47 of them (68.1%) were found to carry *Tyr139Cys* mutations with 25 homozygotes and 22 heterozygotes. Details of their sampling locations and number of mutations are summarised in Table 2 and Fig. 2.

**Table 2:** Summary of samples’ location and no. of *Y139C* mutation found in *R. tanezumi*.

District	*Rattus* sp.	Sample no	No. of *Y139C*	%
Central and Western	*R. norvegicus*	3	–	–
	*R. tanezumi*	1	0	0
Eastern	*R. norvegicus*	2	–	–
	*R. tanezumi*	2	1	50
Islands	*R. norvegicus*	4	–	–
	*R. tanezumi*	7	6	85.7
Kowloon City	*R. norvegicus*	2	–	–
	*R. tanezumi*	1	1	100
Kwai Tsing	*R. norvegicus*	2	–	–
	*R. tanezumi*	4	4	100
Kwun Tong	*R. norvegicus*	2	–	–
	*R. tanezumi*	8	8	100
North	*R. norvegicus*	15	–	–
	*R. tanezumi*	1	1	100
Sai Kung	*R. norvegicus*	2	–	–
	*R. tanezumi*	10	2	20
Sha Tin	*R. norvegicus*	2	–	–
	*R. tanezumi*	4	3	75
Sham Shui Po	*R. norvegicus*	2	–	–
	*R. tanezumi*	1	1	100
Southern	*R. norvegicus*	5	–	–
	*R. tanezumi*	1	0	0
Tai Po	*R. losea*	46	–	–
	*R. norvegicus*	1	–	–
	*R. tanezumi*	11	7	63.6
Tsuen Wan	*R. losea*	6	–	–
	*R. norvegicus*	3	–	–
	*R. tanezumi*	1	1	100
Tuen Mun	*R. norvegicus*	7	–	–
	*R. tanezumi*	2	2	100
Wan Chai	*R. norvegicus*	1	–	–
	*R. tanezumi*	1	1	100
Wong Tai Sin	*R. norvegicus*	25	–	–
	*R. tanezumi*	6	5	83.3
Yau Tsim Mong	*R. norvegicus*	1	–	–
	*R. tanezumi*	2	2	100
Yuen Long	*R. norvegicus*	2	–	–
	*R. tanezumi*	6	2	33.3
Total	*R. losea*	52	–	–
	*R. norvegicus*	81	–	–
	*R. tanezumi*	69	47	68.1

### Other SNPs on the *VKORC1* gene

Table 3 summarised all the located SNPs obtained from the selected *R. tanezumi* and *R. norvegicus* samples subjected to whole genome sequencing based on their geographical distributions.

**Table 3:** Summary of SNPs located in *R. losea*, *R. norvegicus* and *R. tanezumi*.

Exon	Species	SNP location (DNA)	Alleles	Genotype frequency			Potential mutation
Exon 1	*R. norvegicus*	137	C/A	CC	CA	AA	*Asp44Glu*
				26	1	0	
	*R. tanezumi*	128	G/A	GG	GA	AA	*Ala41Ala*^
				3	10	14	
Exon 2	*R. norvegicus*	209	T/C	TT	TC	CC	*His68His*
				22	0	1	
		250	A/T	AA	AT	TT	*Ile82Ile*
				16	3	4	
Exon 3	*R. norvegicus*	326	C/T	CC	CT	TT	*Ile107Ile*
				70	1	0	
	*R. tanezumi*	438	A/G	AA	AG	GG	*Tyr139Cys*^*#*^^
				21	21	21	
	*R. losea*	293	C/T	CC	CT	TT	*Cys96Cys*
				51	1	0	
		299	A/G	AA	AG	GG	*Arg98Arg*
				51	0	1	
		308	G/T	GG	GT	TT	*Trp101Cys*
				51	1	0	

^#^ known missense mutation.

^ also observed from re-sequenced individuals.

In addition to the known *Y139C* mutation, nonsynonymous mutations were also found from one *R. norvegicus* sample and one *R. losea* sample, respectively. Further, six synonymous mutations were also found among three species. Details are provided in Supplementary information S1.

Besides the exons, a total of nine SNPs was revealed locating at the introns, with three coming from *R. norvegicus* and the other six from *R. tanezumi* (Table 4).

**Table 4:** Summary of SNPs locating at introns of *VKORC1* gene.

Sample name	Reference sequence	Position	Nucleotide changed
Is_1 (*R. norvegicus*)	NC_005100.4	199,340,196	T → A/T
		199,340,007	A → A/T
		199,339,548	T → C/T
YL_2 (*R. tanezumi*)		199,341,071	C → T
		199,340,872	T → C
		199,340,543	A → G
		199,339,540	G → A
		199,339,461	C → A
		199,338,993	A → G

### Ethics declaration

Animal ethics approval was granted by the Animal Research Ethics Sub-Committee of City University of Hong Kong. All methods were carried out in accordance with relevant guidelines and regulations. All methods are reported in accordance with ARRIVE guidelines.

## Discussion

Efficacy of rodenticides is crucial for effective rodent management, and this study carried out the first *VKORC1-*based survey to estimate the anticoagulant rodenticide resistance situation. In contrast to the previous rodent species identification in Hong Kong revealing eight species of rats and mice, with *Rattus norvegicus* and *R. rattus* to be the dominant rat species in urban areas^3^. This study, nevertheless, identified three *Rattus* species including the report of the *R. losea*, *R. norvegicus*, and *R. tanezumi* based on molecular marker *COX1*. The number of captured rats has revealed the abundance of *R. tanezumi* and *R. norgevicus*, while the *R. losea* were captured from two locations only. Despite *R. rattus* and *R. tanezumi* were well known to be difficult to be morphologically differentiated from one another^22,25^, given the previous and present studies were carried out at different time (more than ten years) and places using different collection method, it is unclear whether the situation represents misidentification, distribution in different biotopes, different collection methods, or changes in dominant rodent species spatiotemporally.

In the limited studies carried out on anticoagulant rodenticide resistance in Asia, a relatively low warfarin-resistance rate (11%, 4 out of 36 samples) was determined by lethal feeding test in *R. tanezumi* collected from mainland China ten years ago^26^. It should be noticed that the use of anticoagulant rodenticides in China was believed to have started in the early 1980s^27^, which has a shorter history than other places in the world. A recent study also suggested a low anticoagulant rodenticide resistance rate in *R. norvegicus* collected from two cities in mainland China^28^. This study, based on the *VKORC1* gene survey, discovered 68.1% of *R. tanezumi* in Hong Kong carried the *Tyr139Cys* mutation.

Previous studies suggested that the *Tyr139Cys* mutation could confer resistance to first- and second-generation anticoagulant rodenticides including bromadiolone and difenacoum in Norway rat and house mouse^19^. Given the relationships between anticoagulant rodenticide resistance and the *Tyr139Cys* mutation in *R. tanezumi* has not been tested, the cause and significance of such mutation being only observed in *R. tanezumi* but not in *R. losea* and *R. norvegicus* remains to be revealed. In case if the *Tyr139Cys* mutation in *R. tanezumi* also confer certain type of anticoagulant rodenticide resistance, other substances such as difethialone and flocoumafen could to be used^29^. Regarding to the rodent nuisance in Hong Kong, anticoagulant compound is more desirable and safer rodenticide for controlling rodents compared with acute poison within the densely populated urban area. Anticoagulant compound has been widely adopted by both private and public pest control operators. Currently, there is no statutory regulation to monitor the use of rodenticide from local pest control operators, however, the low efficacy of certain compound and good prevention practice should be aware in order to decrease the influence of rodent problems.

This study also revealed other SNP variants not documented previously, for instances, two synonymous SNPs and one nsSNP (*Trp101Cys*) in *R. losea*. It is also worth noting that no SNPs located in exon 1 identified from the nine *R. losea* samples were *Arg58Gly* mutation which confer anticoagulant rodenticide resistance^30^. These data bring up the issues that there are huge gaps in knowledge regarding the origin, introduction, genetic diversity, population connectivity of *Rattus* between different places in Asia, as well as the relationships of mutations brought in to *VKORC1* genes and their anticoagulant rodenticide resistance of different *Rattus* population from different places in Asia.

## Conclusion

This study provided the baseline information of rodenticide resistance status and distribution of 202 rodents belonging to 3 *Rattus* species in Hong Kong. The investigation indicates a distinctive anticoagulant rodenticide resistance pattern. The relatively high *Tyr139Cys* mutation found in *VKORC1* gene of *R. tanezumi* suggested further susceptibility tests will be needed to reveal whether they are resistance to individual anticoagulant rodenticide and to ensure effectiveness on local species. Regular genetic testing and genomic analyses will also be required to understand the situations of rodent populations for integrated pest management.

## Supplementary Information

Supplementary Information.

## Data Availability

The raw reads generated in this study have been deposited to the NCBI database under the BioProject accession PRJNA723168.
